# Supplementary material for: SNA077, an Extract of Marine Streptomyces sp., Inhibits Melanogenesis by Downregulating Melanogenic Proteins via Inactivation of cAMP/PKA/CREB Signaling
Source: Int J Mol Sci. 2022 Nov 29;23(23):14922. doi: 10.3390/ijms232314922 (PMC9737552; doi:10.3390/ijms232314922)

Supplementary Figures

Figure S1. UV spectra of prodigiosin in SNA077 fraction (blue) in comparison with in-house known compound (red). (A) prodigiosin, match score = 998.

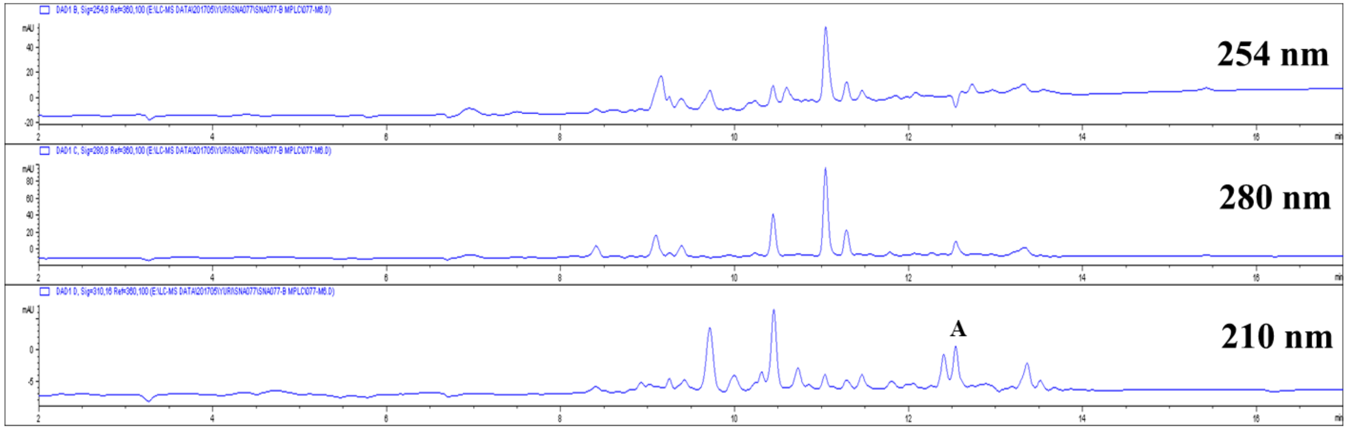

A

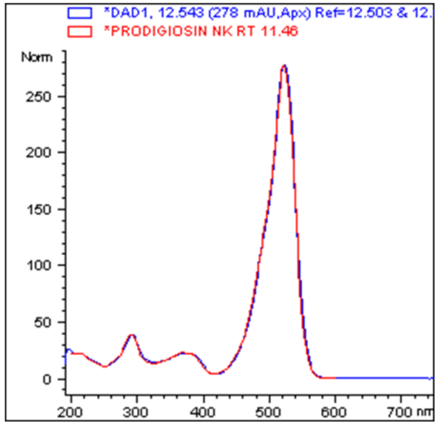

Figure S2. LRMS Data of germicidin A

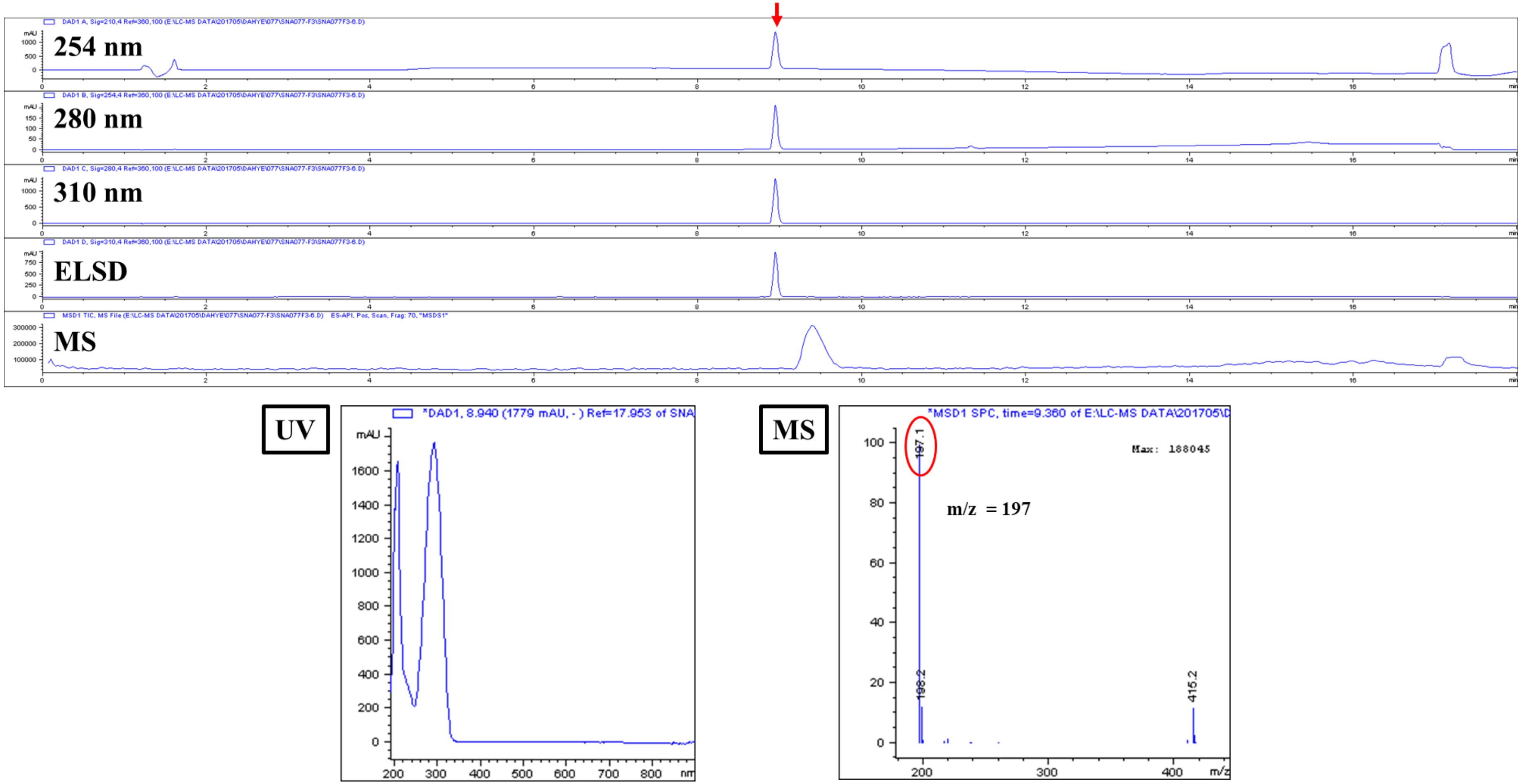

Figure S3. ^{1}H NMR Spectrum of germicidin A in CD_3OD

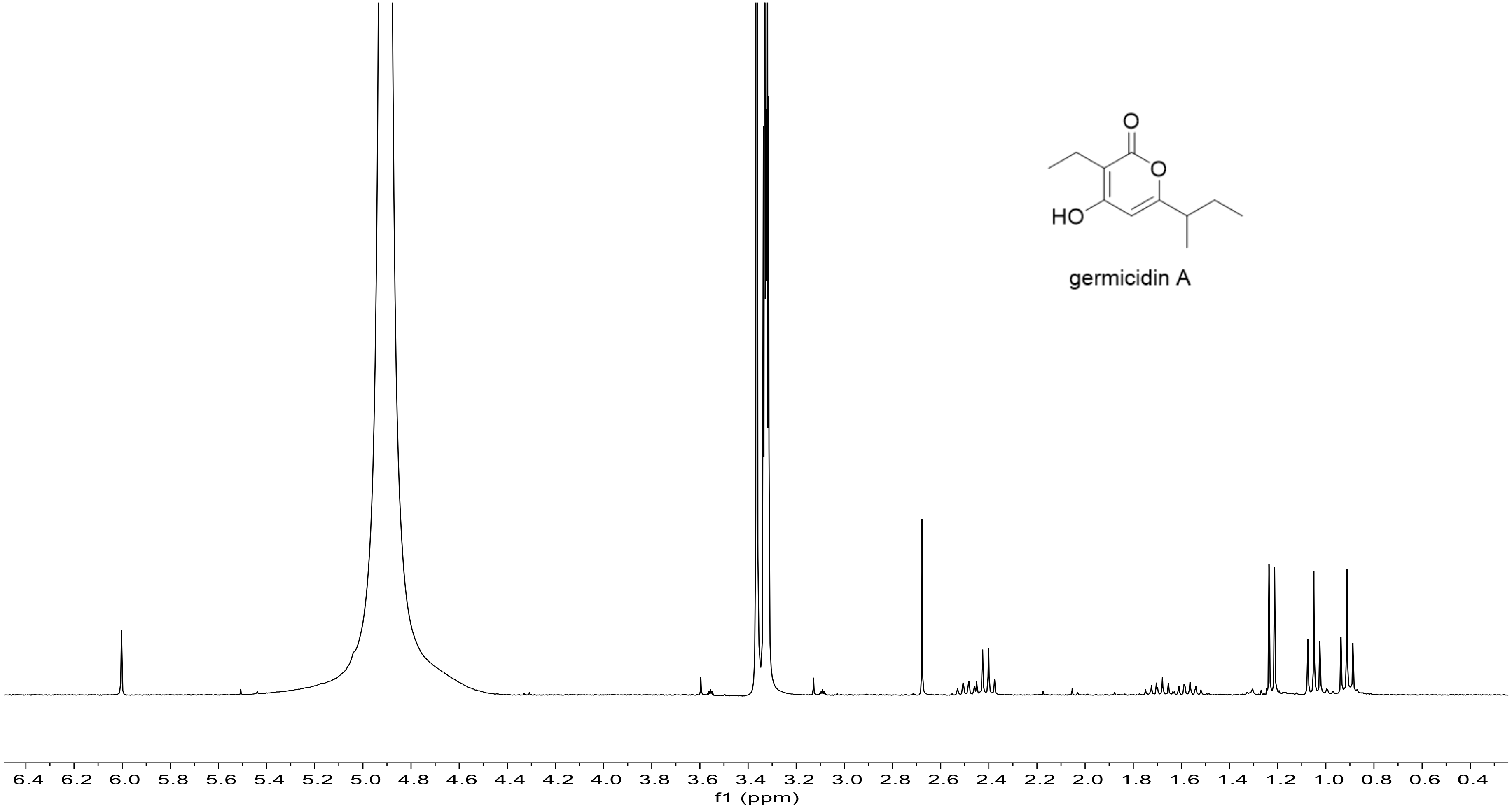

Figure S4. ^{13}C NMR Spectrum of germicidin A in CD_3OD

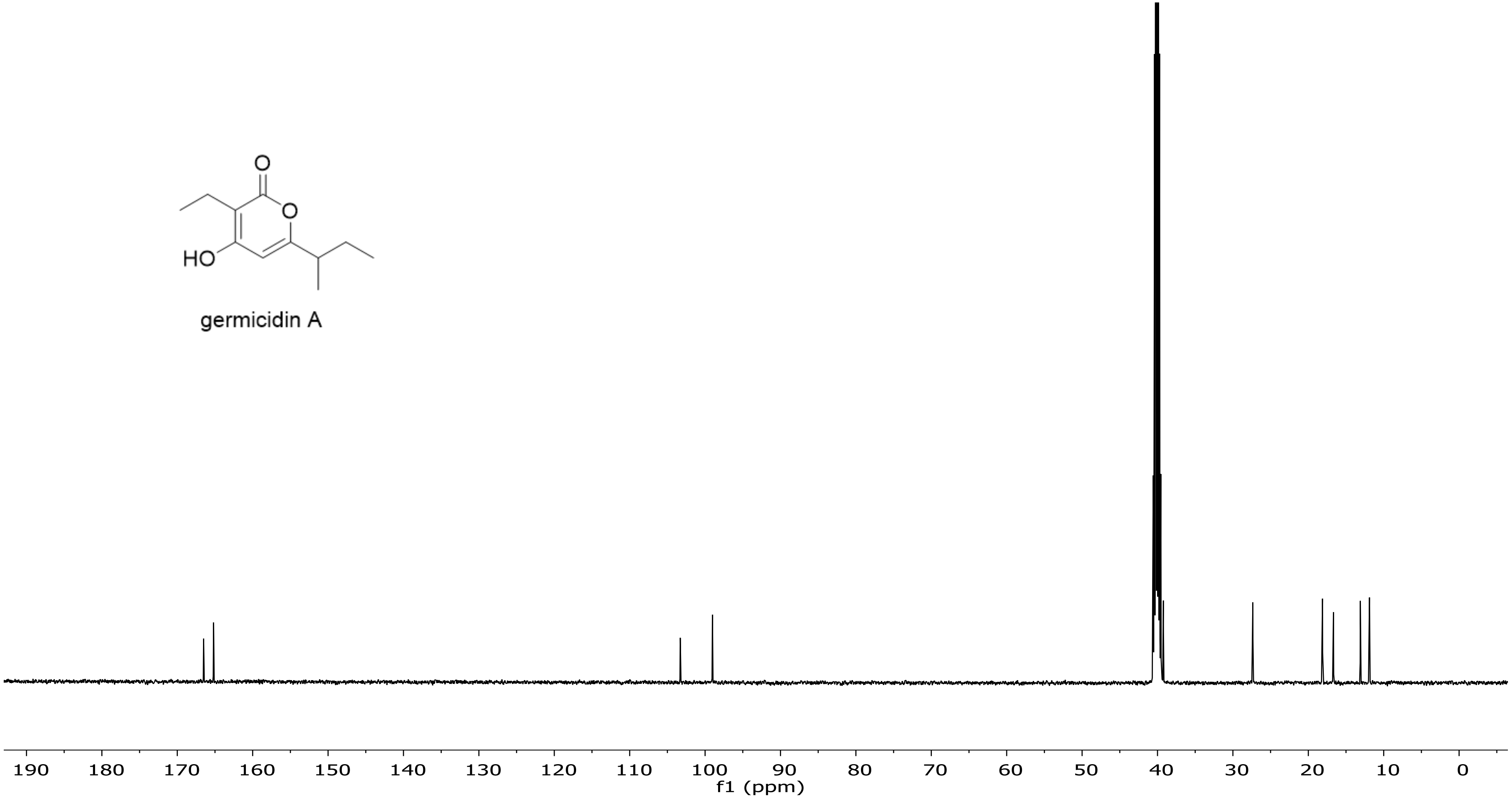

Figure S5. LRMS Data of Isogermicidin A

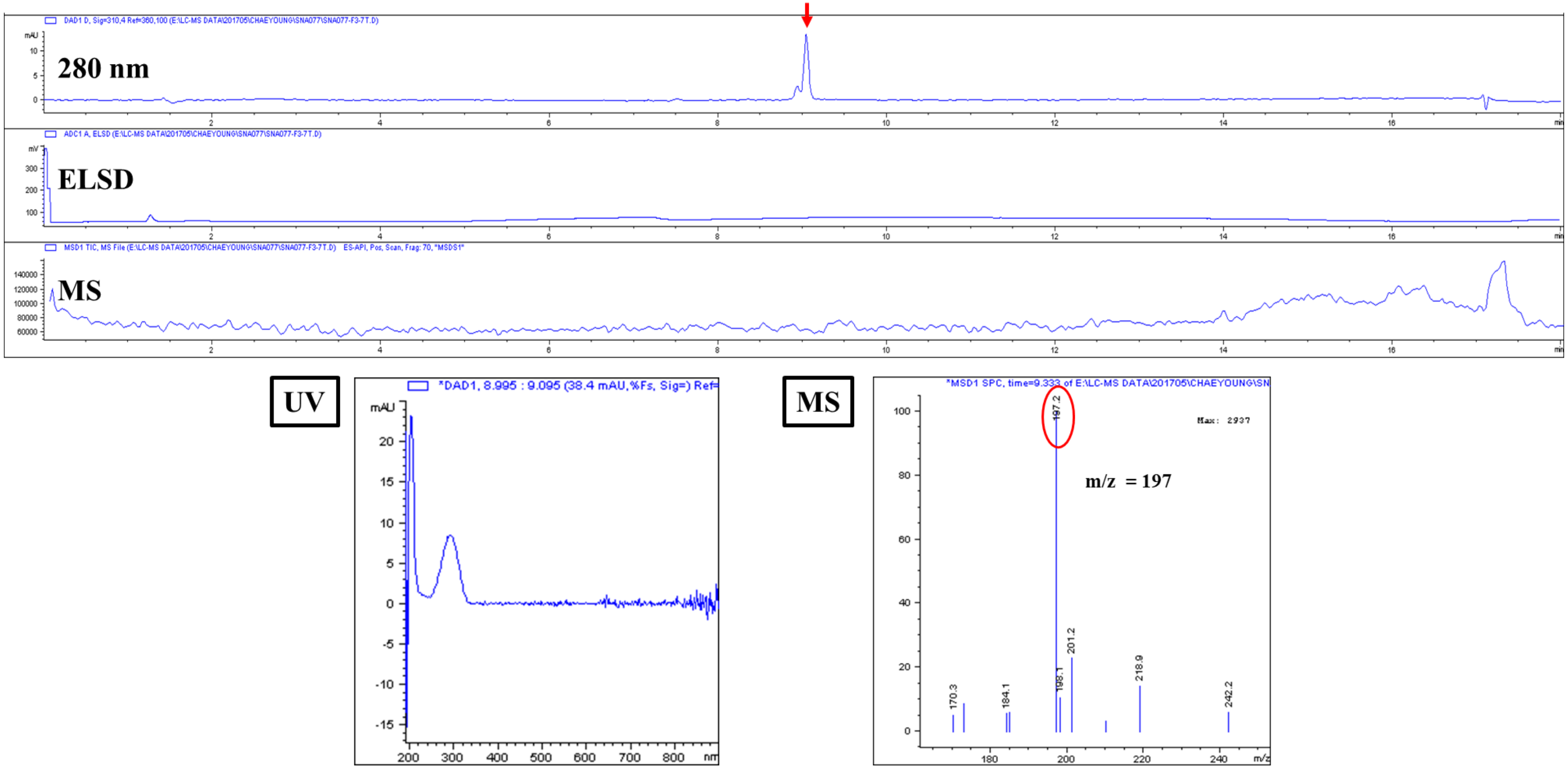

Figure S6. ^{1}H NMR Spectrum of Isogermicidin A in CD_3OD

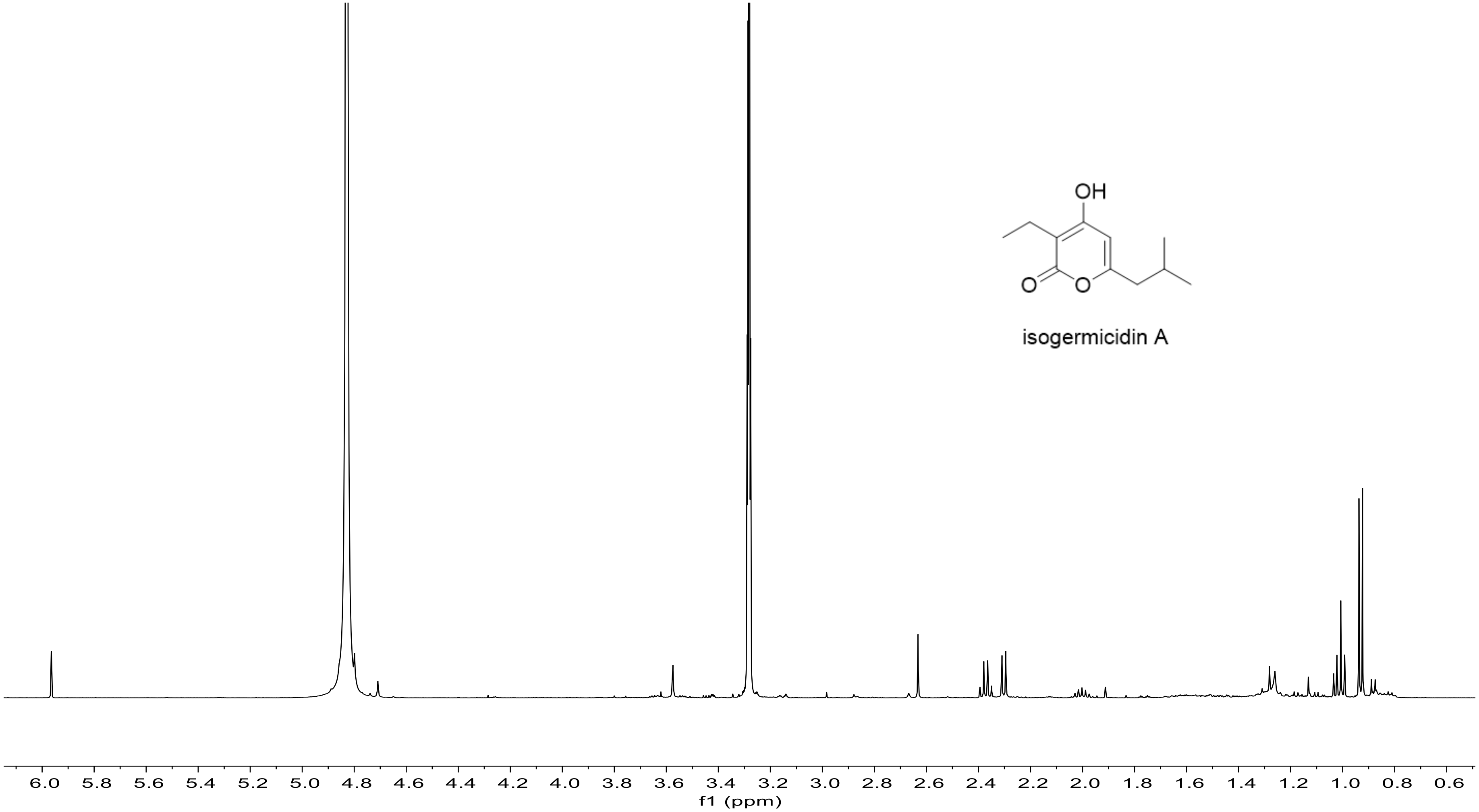

Figure S7. ^{13}C NMR Spectrum of Isogermicidin A in CD_3OD

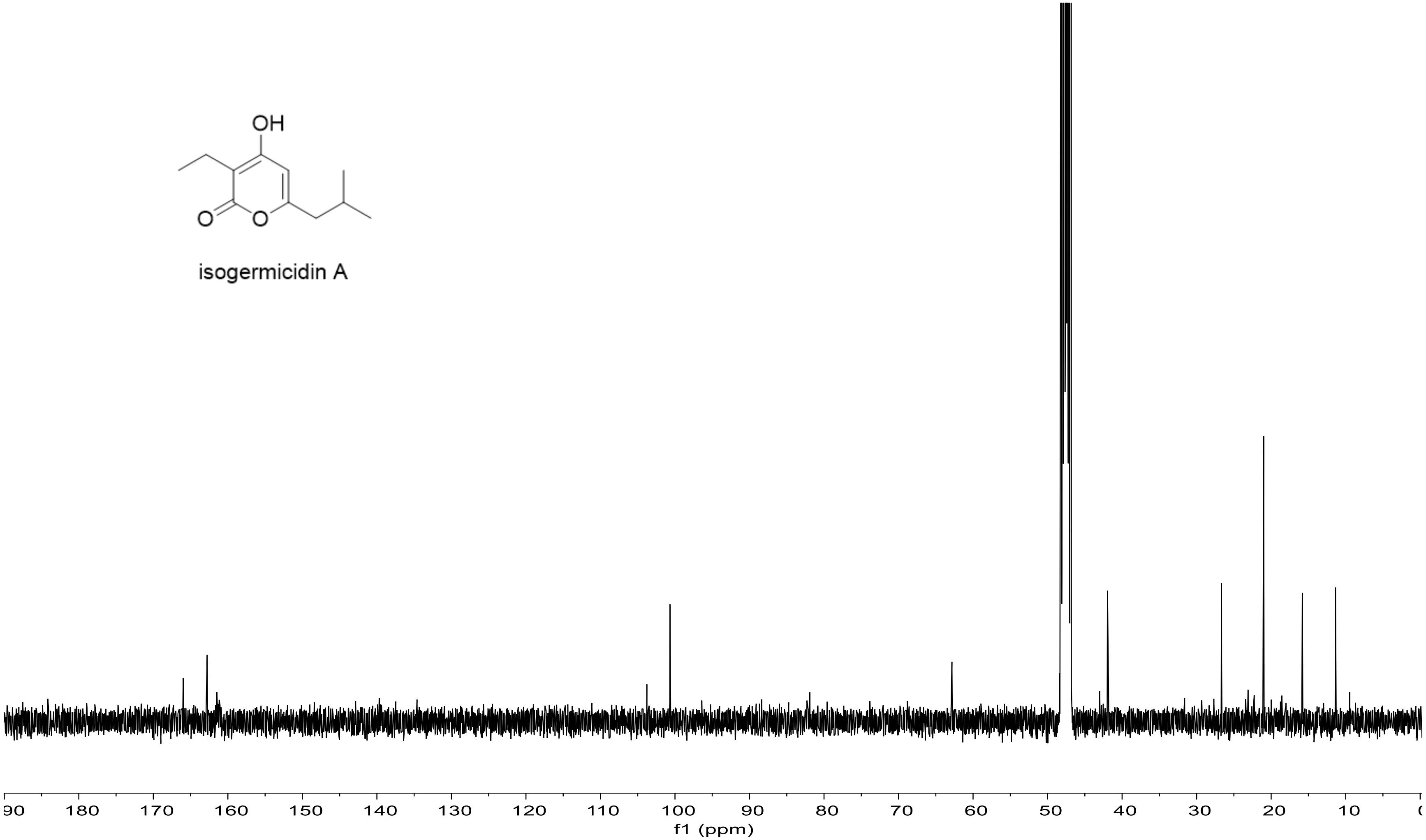

Figure S8. LRMS Data of Prodigiosin

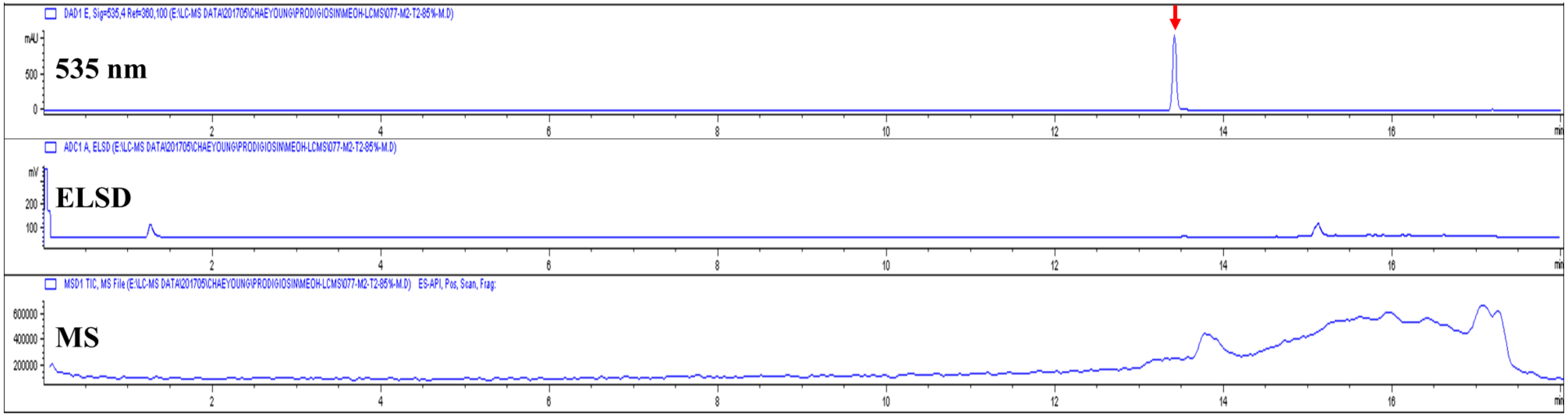

UV

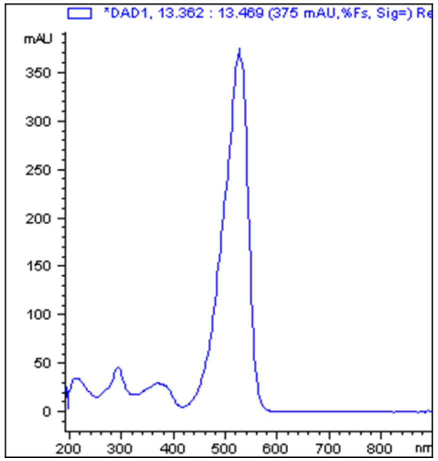

MS

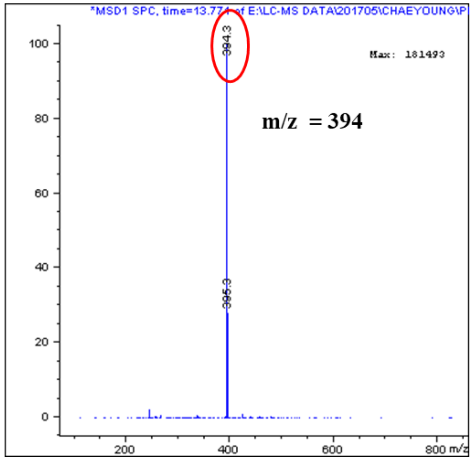

Figure S9. ^{1}H NMR Spectrum of Prodigiosin in $CDCl_3$

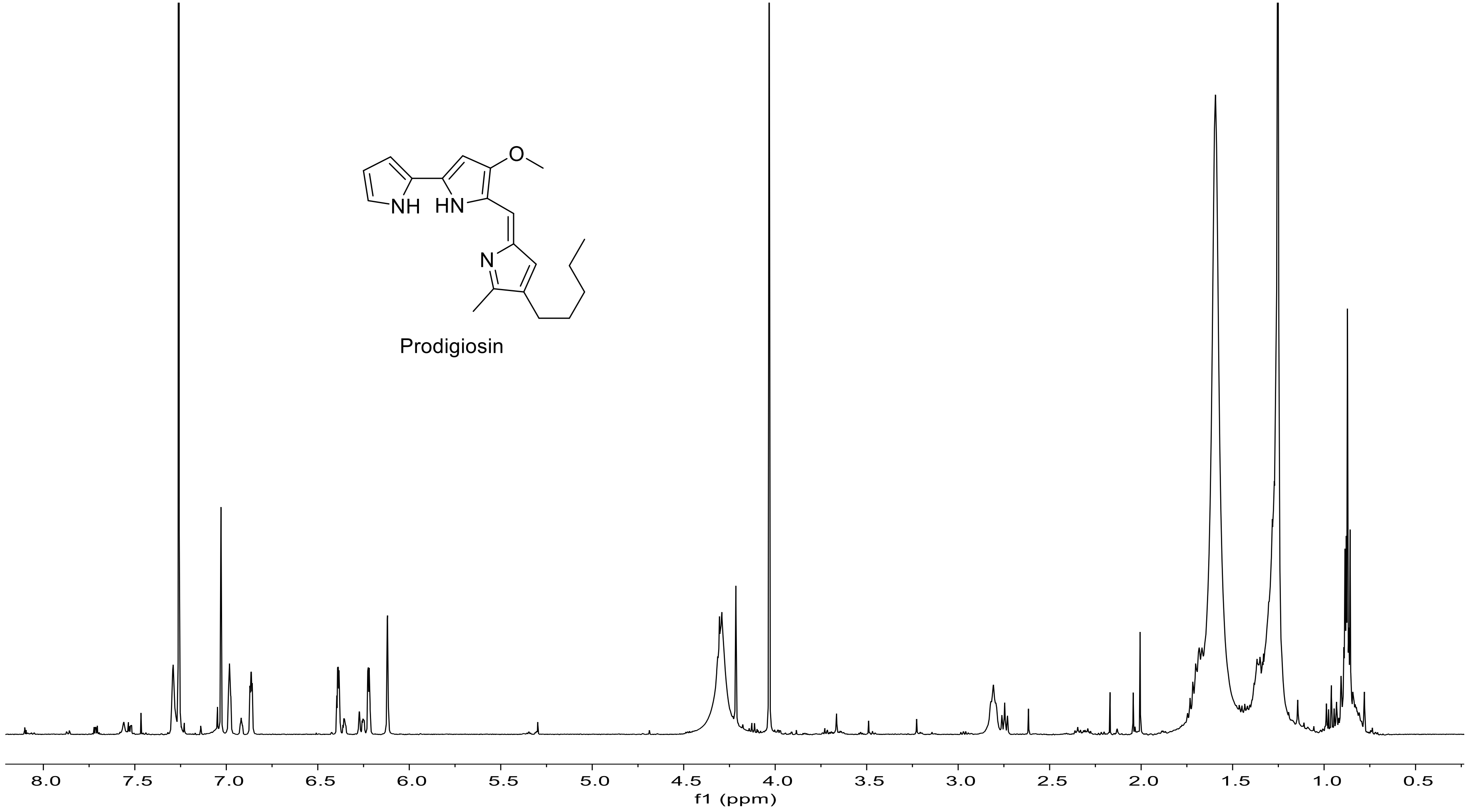

Supplement: Supplementary file 1 [file ijms-23-14922-s001.zip › ijms-2034592-supplementary.pdf]
